# Symptoms of Diminished Autonomy over Cigarettes with Non-Daily Use

**DOI:** 10.3390/ijerph6010025

**Published:** 2008-12-23

**Authors:** Judith A. Savageau, Paul D. Mowery, Joseph R. DiFranza

**Affiliations:** 1 Department of Family Medicine and Community Health, University of Massachusetts Medical School, 55 Lake Avenue, Worcester, Massachusetts 01655, USA; E-Mail: judith.savageau@umassmed.edu; 2 Biostatistics, Inc., Atlanta, Georgia, USA; E-mail: moweryp@bellsouth.net

**Keywords:** Smoking, adolescents, tobacco, dependence, addiction

## Abstract

Data from a nationally representative sample of smokers (ages 12–22 years, n=2,091) was examined to investigate the prevalence of symptoms of diminished autonomy over cigarettes. Six symptoms were assessed: failed cessation, smoking despite a desire to quit, and a need or urge to smoke, irritability, restlessness, or disrupted concentration attributed to nicotine withdrawal. One or more of the six symptoms were present in 18.9% of subjects who smoked less often than once per week. Among subjects who had not smoked 20 cigarettes in their lifetime, 12.6% had symptoms of nicotine withdrawal, and 25% had made an unsuccessful quit attempt.

## 1. Introduction

Early workers in the field of nicotine addiction believed that a person didn’t become an addicted smoker until he or she smoked many cigarettes each day and had smoked for a number of years [1]. However, adolescents have complained of difficulties with quitting after smoking only one or two cigarettes [2]. To investigate this phenomenon, the Development and Assessment of Nicotine Dependence in Youth studies (DANDY-1 and DANDY-2) followed 6^th^ and 7^th^ grade students over several years [3–6]. The Diagnostic and Statistical Manual of Mental Disorders (DSM) provides criteria that can be used to diagnose “cases” of nicotine dependence, but it does not provide a nomenclature to describe smokers who have one or two symptoms of nicotine dependence, but fall shy of the three criteria cutoff needed for a diagnosis under the DSM rubric [7]. Therefore, studies of smoking onset have only rarely used the DSM criteria [8]. Such studies more commonly assess diminished autonomy over tobacco use. As defined, an individual experiences diminished autonomy when quitting requires an effort or produces discomfort [9]. It should be noted that the appearance of diminished autonomy does not signify a DSM diagnosis of nicotine dependence; but it does represent an indication of a process that may progress to a DSM diagnosis of nicotine dependence.

The 10-symptom Hooked on Nicotine Checklist (HONC) is a validated measure of diminished autonomy, currently in use around the world in 14 languages [9, 10]. In the DANDY studies, each of the following 10 HONC symptoms was reported by at least one subject within two weeks of smoking at least once per month: strong need, urge or craving to smoke, difficulty refraining from smoking, feeling addicted, and anxiety, restlessness, irritability, and difficulty concentrating attributed by the subject to abstinence from tobacco. Each symptom was reported by some youth who had never been daily smokers [5]. Among the subjects who developed these symptoms, 10% had done so within 2 days, and 25% within 30 days of first inhaling from a cigarette [6].

All published studies of the onset of nicotine dependence in the US have involved local convenience samples. The Centers for Disease Control published a brief report of a representative national survey that assessed six symptoms of diminished autonomy in youth, but the data were never analyzed to determine how many youth had one or more of symptoms [11]. This publication represents the first examination of the prevalence of symptoms in relation to the frequency of smoking in a national US sample.

## 2. Methods

The data for this analysis were obtained from the 1993 Teenage Attitudes and Practices Survey (TAPS-II), which was developed under the direction of the National Center for Health Statistics and the Office on Smoking and Health, Centers for Disease Control and Prevention[11–13]. Subjects in the TAPS-II survey were recruited from a probability sample of 5,590 persons aged 10–15 years, and 9,135 respondents to the 1989 TAPS-I survey (aged 12–18 years). The TAPS-I sampling frame consisted of all persons aged 12–19 years who resided in households interviewed for the National Health Interview Survey (NHIS), an annual household interview survey of the non-institutionalized civilian population of the United States. The NHIS uses a multistage sampling design employing both clustering and stratification by race, sex and age [14]. Only the TAPS-I subjects who had been interviewed by phone were eligible for follow-up in TAPS-II. The TAPS-II survey was administered through telephone interviews, or if that was not possible, in person.

The TAPS-II survey included six items that have subsequently been validated and used as indicators of diminished autonomy [9]. “I smoke because it is really hard to quit.” “How many times have you tried to quit smoking?” A prior attempt to quit smoking among current smokers was considered an indicator of diminished autonomy. “When you (quit/tried to quit) smoking did you ... feel a strong need or urge to have a cigarette? ... feel more irritable? ... find it hard to concentrate? ... feel restless?” Response options were yes/no/don’t know/don’t remember, which we recoded as yes/no. We created dichotomous indicators of diminished autonomy (DA; any of the six symptoms including the four withdrawal symptoms versus none), and withdrawal symptoms (any of the four withdrawal symptoms versus none). Demographic data included age, gender, and race. Current smoking was defined as having smoked any cigarettes during the 30 days preceding the survey. Smoking variables (asked as continuous variables) included lifetime cigarettes smoked (which we grouped as <20, 20–99, and 100 or more); the number of smoking days per month (grouped as 1–3 days, indicating less than weekly smoking; 4–29 days, indicating weekly but not daily smoking; and all 30, indicating daily smoking); and the number of cigarettes smoked per day (grouped as 0–4, 5–9, 10–14, and 15 or more). These groupings were chosen to allow for comparisons with other published studies. Only subjects who had smoked during the previous week were asked about the number of cigarettes smoked per day.

Only current smokers were asked the six DA items; thus, our analyses were limited to that population. Further, only smokers who had failed at a quit attempt were asked about withdrawal symptoms. The CDC’s initial publication of the TAPS data therefore used the population of smokers who had failed to quit as the denominator for their prevalence estimates [11]. We felt that this inflated the prevalence estimates because the population was biased to include the most dependent individuals. For our analyses, we conservatively assumed that current smokers who had not been asked the withdrawal questions would have answered no to these items. The number of current smokers was the denominator for our prevalence estimates, which are about 30% lower than those published by the CDC [11].

The brief report published by the CDC included only simple frequency distributions with no further analyses [11]. We used Chi-Square analysis to compare the prevalence of symptoms for subjects with different levels of tobacco use and gender. We performed a series of logistic regressions using the dichotomous outcome of any DA symptoms versus none, controlling for age, gender and race (white versus all others). Independent variables included daily use (no/yes), lifetime cigarette consumption (<20, 20–99, and ≥ 100 cigarettes), smoking days per month (<4, 4–29, and 30), and average daily cigarette consumption (<5, 5–9, 10–14, and ≥ 15 cigarettes). As the measures of tobacco use were correlated, each was entered into a separate regression because of multi-colinearity. Parallel regressions were performed with the dependent variable being withdrawal symptoms (‘any’ versus ‘none’).

Linear regressions were also performed with DA as a continuous measure (number of items endorsed, 0–6) while controlling for age, gender and race. Independent variables entered in separate runs included daily use (no/yes), and as continuous variables: lifetime cigarette consumption, smoking days per month, and average daily cigarette consumption. These regressions were repeated with withdrawal as a continuous variable (0–4 items endorsed) as the dependent variable. A p-value of <0.05 was used as a test of statistical significance. All analyses were weighted to provide estimates for a nationally representative population, and our analyses adjusted for both the clustering and stratified sampling design using SAS V9.1.3 (Proc SurveyReg and Proc SurveyLogistic; SAS Institute, Inc., Cary, NC).

## 3. Results and Discussion

Of the probability sample of 5,590 individuals, 4,992 participated (89.3% response rate). Of the 9,135 subjects followed up from TAPS-I, 7,960 (87.1%) participated in TAPS-II, for a total of 12,952 subjects in TAPS-II. Of these, 51.2% were male and the mean age was 16.0 years (range 10–22, standard deviation 3.5). The sample is described in Table 1. Current smoking was reported by 2,091 subjects (16.2%), of whom 52.9% were male and the mean age was 18.3 years (range 10–22). These subjects are the focus of the current analyses. Of the probability sample of 5,590 individuals, 4,992 participated (89.3% response rate).

All six DA symptoms were reported by 13.2% of current smokers; five by 12.4%; four by 10.3%; three by 8.8%; two by 8.7%; one by 17.6%; and none by 29.1%. Thus, 70.9% of current smokers had at least one symptom. All four withdrawal symptoms were reported by 15.8% of current smokers; three by 12.8%; two by 11.2%; one by 10.4%; and none by 49.8%. Among 35 subjects who had smoked fewer than five cigarettes in their lifetime, 3 (9%) reported a withdrawal symptom, and 35% reported at least one of the six DA symptoms.

**Figure 1:**
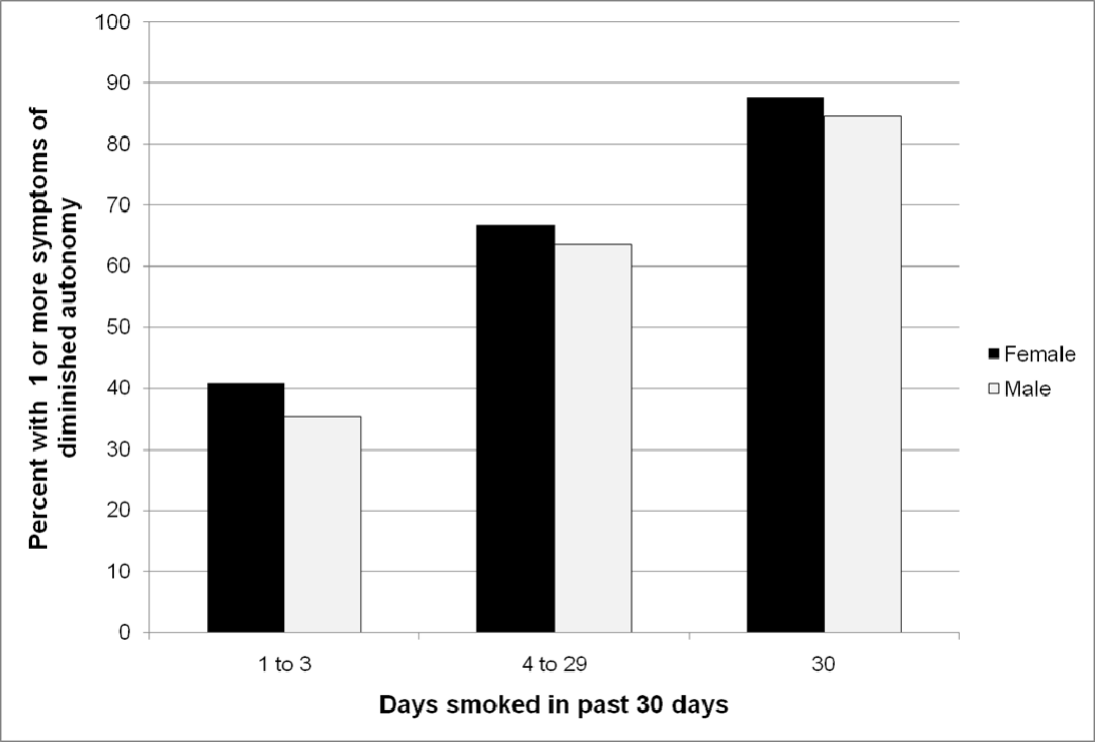
The percent of male and female current smokers reporting any symptom of diminished autonomy in relation to the number of smoking days per month.

Figure 1 and Table 2 present the proportion of current smokers who had DA or withdrawal symptoms in relation to gender and measures of tobacco use. Each symptom was present in subjects at the lowest level for each measure of tobacco use. Females had more symptoms than males. As expected, both DA and withdrawal increased in prevalence in relation to each measure of tobacco use. These relations persisted when controlled for age, gender and race, and when the outcome measure was treated as either a dichotomous or continuous variable (Tables 3 and 4).

These data from a national sample of adolescents and young adults confirm that the earliest symptoms of diminished autonomy can appear after the first few cigarettes. Symptoms were reported by 35% of youth who had smoked fewer than five cigarettes. These data are in agreement with other recent studies. The Natural History of Nicotine Dependence (NDIT) study followed adolescents prospectively over five years to replicate the DANDY-1 study[15, 16]. More than a third of smokers had withdrawal symptoms prior to smoking four cigarettes. Kandel et al. evaluated new adolescent tobacco users for nicotine dependence symptoms using the American Psychiatric Association’s diagnostic criteria [7, 8, 17]. Thirty-five percent of the adolescents who experienced a symptom of dependence had done so within their first month of tobacco use, corresponding well to DANDY-2, in which 25% of those who had experienced symptoms of diminished autonomy did so within one month of initiating use [6]. A New Zealand national survey of almost 30,000 adolescent smokers reported that 27% of current smokers who had smoked only two cigarettes in their lifetime had symptoms of diminished autonomy as did 35% of those who had smoked 3–4 cigarettes [18]. A South African study of adolescents reported that almost half of youth who smoked less than one cigarette per week reported more than two Diagnostic and Statistical Manual nicotine withdrawal symptoms [19]. A 16-year longitudinal study in the US demonstrated that the trajectory to dependent adult smoking was set by the time youth were smoking two cigarettes per week [20]. Our study adds to a growing body of literature that indicates that approximately one-quarter to one-third of young smokers have early symptoms of emerging dependence by the time they have smoked five cigarettes. It should be emphasized that few of these individuals would have met the DSM case criteria for nicotine dependence.

Particularly relevant for clinicians is the impact that the rapid onset of symptoms has for their patients: one-quarter of subjects who had not yet smoked 20 cigarettes reported having already failed an attempt to quit. Some symptoms were already present in 18.9% of subjects who were smoking less often than once per week, and in 29.8% of subjects who had not yet smoked 20 cigarettes. Although these figures may seem high, they likely underestimate the true prevalence of symptoms in this population. In the New Zealand study, 52% of youth who smoked less often than once per week had a symptom of diminished autonomy, as did 42% of subjects who had smoked fewer than 20 cigarettes in total[18]. The prevalence of symptoms we report here is low in comparison to those seen in all previous studies. This is likely attributable to a methodological limitation of the TAPS-II survey; both DANDY studies, the NDIT study and New Zealand study included 10 symptoms of diminished autonomy, but the TAPS-II survey included only six. Importantly, craving outside of a quit attempt was not assessed, and this is the most common presenting symptom and the most prevalent symptom overall[5]. Also, the prevalence data reported here may be low because of our decision to assume that withdrawal symptoms were absent in current smokers who had never failed an attempt to quit smoking. For these reasons, the current study may underestimate the true prevalence of symptoms.

Our data indicate that 16% of subjects who smoked less often than once per week reported withdrawal symptoms. These data likely underestimate the true prevalence of withdrawal symptoms in these very light smokers as only subjects who had tried to quit were asked the four withdrawal questions. Subjects who do not attempt to quit can still experience withdrawal symptoms between cigarettes and would have been missed.

When DANDY-1 reported symptoms of emerging dependence after the first cigarette, the reliability of adolescent reports and the 10 HONC items used to assess diminished autonomy were questioned. As the six items used in the current study to assess diminished autonomy were later incorporated into the HONC, this reliability issue pertains to the current study as well. Conversely, evidence concerning the reliability of the HONC reflects on the reliability of the TAPS-II data. The HONC has demonstrated excellent internal consistency (α = 0.90 – 0.94) in three adolescent studies [9, 10, 21]. Endorsement of one or more HONC item was associated with an odds ratio of 195.8 for progression to daily smoking among subjects who had puffed on a cigarette, and with an odds ratio of 83 among those who had inhaled [6]. The strong predictive power and high internal consistency of the HONC [9, 10, 21], its very good test-retest reliability [10], its stable single-factor structure [10], its ability to predict long-term smoking cessation outcomes [6], and its external validity as demonstrated by associations in the expected directions with lifetime maximum cigarette consumption [5], current smoking days per month [10], daily cigarette consumption [10], greatest length of abstinence, use of pharmacological cessation aids, the modified Fagerström Tolerance Questionnaire and the Fagerström Test of Nicotine Dependence [21–23] are individually, and collectively, incompatible with the hypothesis that adolescents are misreporting symptoms. The dose-response relationship observed between the prevalence of diminished autonomy and each of three measures of tobacco use in the current study provides additional reassurance that symptom reports are reliable.

By documenting the appearance of withdrawal symptoms in youth who have smoked only a few cigarettes, this study adds to the accumulating evidence [4, 6, 16, 18, 19] that contradicts the assumption that nicotine withdrawal is the product of chronic moderate daily use, or that the development of withdrawal symptoms requires a person to smoke at least five cigarettes per day to remain comfortable. As demonstrated by the data in this study, withdrawal symptoms can appear while cigarettes are spaced more than a week apart. Adolescents describe how withdrawal symptoms can be initially kept at bay for a week or more by smoking a single cigarette. But over time, the duration of relief obtained from each cigarette shortens progressively and youths must space their cigarettes at ever closer intervals to suppress withdrawal [24].

Do novice smokers experience withdrawal the same as adult smokers? When youth report difficulty concentrating, are they experiencing the same thing as an adult who reports difficulty concentrating? (One might also question whether two adult smokers are having the same experience.) The relevant question is not whether youths’ symptoms are identical to those of adults, but whether adolescent symptoms affect the trajectory of smoking. They do, the appearance of symptoms increases the risk of progressing to daily smoking by nearly 200-fold [6]. Whatever it is that youth are experiencing when they report symptoms after smoking one or two cigarettes, it has great clinical relevance.

Strengths of this study include the relatively large nationally representative sample, high participation rate, wide age range, racially mixed population, and the use of the diminished autonomy measure that allows us to compare this dataset to those from the two DANDY studies, NDIT and the New Zealand national survey which all used this metric. This report adds to the previous brief report of this dataset by (1) using all smokers as the denominator, (2) calculating the percentage of subjects who had one or more symptoms of diminished autonomy to determine the proportion of youth who are affected at each level of tobacco use, and (3) including logistic and linear regression analyses to determine the impact of gender, current use, and lifetime tobacco use on the emergence of diminished autonomy and withdrawal symptoms. Limitations include the use of only six symptoms of diminished autonomy. Nicotine dependence as defined by the DSM was not assessed, although the symptoms we evaluated are considered symptoms of nicotine dependence according to the DSM criteria[7]. We relied on symptom self-reports as the symptoms of nicotine dependence are largely subjective. A recent study demonstrates that nicotine dependence symptom reports by adolescents cannot be attributed to false reporting based on expectations [25]. Although the data were collected a number of years ago, the relationship between tobacco use and symptom development would not have changed.

## 4. Conclusions

This national study adds to the mounting evidence that symptoms of diminished autonomy, including those of nicotine withdrawal, can appear after the first few cigarettes, and appear commonly in youth who are non-daily smokers.

## Figures and Tables

**Table 1:** Sample characteristics.

	n	%

**Gender**		

Male	6636	51.2
Female	6316	48.8

**Age**		

10–14	4691	36.2
15–17	3360	25.9
18–19	2269	17.5
20+	2632	20.3

**Race**		

White	10254	79.2
Non-white	2698	20.8

**Smoking status among ever smokers**		

Not a current smoker	2380	53.2
Current smoker	2091	46.8

**Lifetime number of cigarettes smoked (smokers only)**		

<20	1531	35.0
20–99	849	19.4
100+	1996	45.6

**Days smoked in past 30 days (smokers only)**		

0	2380	53.2
1–3	384	8.6
4–29	628	14.0
30	1079	24.1

**Average number of cigarettes smoked per day (smokers only)**		

0–4	575	34.5
5–9	313	18.8
10–14	310	18.6
15+	469	28.1

**Table 2:** The prevalence of symptoms of diminished autonomy in relation to gender and current and lifetime tobacco use. The cells indicate the percentage of subjects within each horizontal category reporting the symptom or symptoms listed at the head of each column. The Chi Square test comparing the prevalence of each symptom for the different categories within lifetime use, smoking days, cigarettes/day and gender were all significant at p<0.0001 (n=2,091).

	Diminished Autonomy (any symptom)	Any withdrawal	Previous quit attempt	Really hard to quit	Strong need or urge	Feel more irritable	Feel restless	Hard to concentrate

**Total population**	70.9†	50.2	62.1	51.3	41.7	37.3	32.4	23.0

**Lifetime cigarette use**								

1–19 (7.5%†)	29.8	12.6	24.9	6.8	4.9	7.8	5.5	5.4
20–99 (14.2%)	42.5	22.4	42.0	13.9	15.3	14.0	9.4	7.1
100+ (78.3%)	80.6	59.7	69.3	62.8	50.8	44.9	39.7	27.8

**Smoking days per month**								

1–3 (18.9%)	37.5	15.9	32.5	10.3	9.8	9.8	8.7	5.3
4–29 (29.8%)	65.6	47.2	59.7	40.9	36.3	29.7	28.5	18.1
30 (51.3%)	86.5	64.9	73.9	72.4	56.9	52.1	43.6	32.6

**Cigarettes smoked per day**								

0–4 (34.4%)	59.9	39.0	55.3	33.6	29.6	23.8	22.8	13.8
5–9 (18.8%)	81.4	58.9	71.2	63.2	51.2	44.7	36.2	24.8
10–14 (18.3%)	87.7	68.0	76.8	74.7	59.3	54.5	45.8	31.2
15+ (28.5%)	90.2	68.9	76.1	78.8	61.0	56.2	49.8	38.7

**Gender**								

Males (51.2%)	68.9	48.3	60.0	51.2	40.2	34.6	30.8	21.2
Females (48.7%)	73.1	52.3	64.4	51.4	43.3	40.2	34.2	25.0

^†^ Percent of total population of current smokers.

**Table 3:** Relationship of diminished autonomy and tobacco withdrawal symptoms to four measures of tobacco use: logistic regression controlling for age, gender and race.

	Diminished autonomy		Withdrawal	
	OR	CI	OR	CI
**Ever smoked daily**				
No	1.00		1.00	
Yes	7.04	5.63–8.81	6.63	5.26–8.35
**Lifetime cigarette use**				
<20	1.00		1.00	
20–99	2.22	1.18–4.15	2.19	1.17–4.10
100+	12.42	6.87–22.45	12.01	6.68–21.61
**Smoking days per month**				
<4	1.00		1.00	
4–29	4.98	3.52–7.03	4.89	3.47–6.88
30	10.30	7.35–14.43	10.02	7.20–13.96
**Cigarettes smoked per day**				
<5	1.00		1.00	
5–9	2.99	2.13–4.20	2.31	1.71–3.12
10–14	4.93	3.32–7.34	3.45	2.47–4.82
15+	6.47	4.61–9.08	3.65	2.81–4.75

**Table 4:** Association of diminished autonomy and tobacco withdrawal symptoms with four measures of tobacco use: linear regression controlling for age, gender and race.

	Diminished autonomy			Withdrawal		
	β	SE	p	β	SE	p
**Ever smoked daily**	2.14	.088	.0001	1.34	.062	.0001
**Cumulative lifetime cigarette use**	0.03	.001	.0001	0.02	.001	.0001
**Smoking days per month**	0.09	.003	.0001	0.05	.002	.0001
**Cigarettes smoked per day**	0.08	.010	.0001	0.05	.007	.0001
